# Predictive heating load management and energy flexibility analysis in residential sector using an archetype gray-box modeling approach: Application to an experimental house in Québec

**DOI:** 10.1177/17442591241267833

**Published:** 2024-09-20

**Authors:** Matin Abtahi, Andreas Athienitis, Benoit Delcroix

**Affiliations:** 1 Department of Building, Civil and Environmental Engineering, Centre for Zero Energy Building Studies, Concordia University, Montréal, QC, Canada; 2 Laboratoire des Technologies de l’Énergie, Hydro-Québec Research Institute, Shawinigan, QC, Canada

**Keywords:** Energy flexibility, optimal load management, model-based predictive control, residential sector, demand-side response, gray-box energy models, time-of-use tariff, dynamic pricing

## Abstract

This paper presents a methodology to develop archetype gray-box models and use them in an economic model-based predictive control algorithm to simulate optimal heating load management in response to a newly-introduced static time-of-use tariff for Québec’s residential sector, rate Flex-D. The methodology is evaluated through a case study, wherein in situ measurements from a two-storey unoccupied research house of Hydro-Québec are used to develop an 11R6C network with a heuristic zoning-by-floor approach and compute the sequence of optimal electric heating input for the next control horizon. Properly-tuned economic model-based predictive control under rate Flex-D shows potential for an approximately 30% reduction in daily heating cost compared to the reference operation, with a minimal average deviation of indoor air temperature from the reference setpoint. Also, the analysis of the response’s sensitivity to weather forecast uncertainties indicates that the most influential uncontrolled input directing the performance of economic model-based predictive control is the structure price signal, rendering the impact of uncertainty in the weather forecast negligible.

## Introduction

Energy flexibility enables demand-side management and helps minimize the stress on the grid as demand peaks. Several national and international initiatives, including the International Energy Agency’s Energy in Buildings and Communities (IEA-EBC) Annex 67 (Jensen et al., 2017), Annex 81 (IEA EBC, n.d.-a), Annex 82 (IEA EBC, n.d.-b), Annex 84 (IEA EBC, n.d.-c), and the Grid-Interactive Efficient Buildings (GEB) program by the U.S. Department of Energy (Satchwell et al., 2021), focused on advancing the theoretical and practical aspects of energy flexibility in buildings.

The IEA-EBC Annex 67 has defined building energy flexibility as “the ability to manage short-term demand and generation according to local climate conditions, the user needs, and grid requirements without jeopardizing the technical capabilities of the operating systems and the comfort of occupants.” (Jensen et al., 2017). Li et al. (2023) have comprehensively reviewed data-driven energy flexibility Key Performance Indicators (KPIs) for buildings in operation, categorizing and analyzing them according to type, complexity, scope, key stakeholders, data requirement, baseline requirement, resolution, and popularity.

Advanced control strategies of heating and cooling systems, specifically Model-based Predictive Control (MPC), are proven to reduce energy consumption and, therefore, operational costs while ensuring occupant thermal comfort (Freund and Schmitz, 2021; Seal et al., 2020; Vallianos et al., 2019, 2023). MPC uses a mathematical model of the building and forecasts of weather and other uncontrolled inputs (disturbances) to predict its future behavior and derive optimal control actions (Drgoňa et al., 2020).

MPC has also shown promise in Demand-side Response (DR) applications, as it exploits the building’s inertia, that is, its thermal and electrical energy storage capacity, to shift consumption and thereby increase energy flexibility (Johra and Heiselberg, 2017; Johra et al., 2019; Murray et al., 2021; Oliveira Panão et al., 2019) or manage transactive interaction with the grid (Denholm and Hand, 2011). Challenges posed to the grids due to the electrification of heating and transport, for example, the growing popularity of heat pumps and electric vehicles, are pushing for the increased application of DR measures (Padovani et al., 2021; Ravi and Aziz, 2022).

The main approaches to building energy modeling are (1) physics-driven (white-box), based on the principles of energy/mass conservation and heat transfer, with a comprehensive description of building geometry, systems, and materials; (2) data-driven (black-box), purely based on statistics and machine learning methods, with no/little information about the building physics and systems; and (3) hybrid (gray-box) approach, based on a simplified description of building physics and systems, with physically-meaningful parameters identified from measurements (Li et al., 2021).

Most simulation tools, like EnergyPlus and TRNSYS, use detailed white-box models and therefore require expertise to create, are time-consuming to calibrate (Abtahi et al., 2022a; Candanedo et al., 2022) and require a great deal of knowledge about the building, which is often not available but assumed (Abtahi et al., 2023a, 2023b). For these reasons, they are not ideal candidates for control applications. On the other hand, black-box models are trained with measured data and do not require the expertise of a building engineer (Maddalena et al., 2020). Trained on enough data, black-box models are highly accurate; however, they cannot often extrapolate to situations not “seen” in the training set or from building to building (Afram et al., 2017). This poses a challenge when using black-box models for optimal operations as they are trained on data from suboptimal control strategies or for specific seasons or operating modes (Afram and Janabi-Sharifi, 2014).

Gray-box models require much less data for calibration (Arendt et al., 2018) and are more likely to stay reliable outside the training range (Afroz et al., 2018) compared to black-box models. Compared to the white-box models, gray-box models are faster to set up and calibrate and require much less prior knowledge/information. Therefore, gray-box models have been repeatedly recognized as the best candidates for short-term optimal decision-making and energy flexibility analysis (Abtahi et al., 2022b; Harish and Kumar, 2016; Wang and Chen, 2019).

A gray-box approach is not an oversimplification of the topology but a selection of relevant information. It requires knowledge of the application and its physics and a coherent choice of significant inputs and outputs. Common simplifications in gray-box models to facilitate the representation of buildings’ thermal dynamics are (1) linearization of heat transfer coefficients, (2) temporal discretization, and (3) reduction of spatial dimensions, also known as zoning (Athienitis and O’Brien, 2015). Essentially, an ideal gray-box model is the simplest one that describes all the dominant patterns and information embedded in the measurements (Bacher and Madsen, 2011). The most common practice in gray-box modeling is the use of thermal Resistance-Capacitance (RC) networks (Athienitis et al., 2015).

The heat balance in thermal RC networks is determined through heat exchange between capacitances and various sources that are either controlled or uncontrolled. Defining the network’s structure, that is, the order (number of capacitances) and the heat exchange paths (number and placement of resistances), is not straightforward. Low/high order affects the model’s structural identifiability (Agbi et al., 2012). An oversimplified model may be unable to adequately capture the thermal dynamics in different zones, whereas a high-order model may be over-parameterized and suffer from parameter redundancy, meaning that there are multiple sets of parameter values that yield the same model (Brastein et al., 2018). The structure of thermal RC networks is defined with either (1) a heuristic or (2) an automated approach.

A heuristic approach entails configuring the structure of thermal RC networks to encapsulate merely one or a limited number of zones, where the determination of the order and dominant heat exchange paths is based on either (1) established zoning approaches in the literature, for example, 1R1C or 3R2C (Fonti et al., 2017; Harb et al., 2016; John et al., 2018; Žáčeková et al., 2014) or (2) observations and control expertise for case-novel structures. As an example of the latter, Abtahi et al. (2022a) compared defining the structure based on the number of floors, that is, zoning by floor (6R3C), and based on the orientation of each room, that is, zoning by orientation (7R3C), as two zoning approaches in residential buildings with smart thermostats; in zoning by floor, each floor is a separate thermal zone represented by a lumped thermal capacitance, whereas, in zoning by orientation, the southern and northern rooms are separate thermal zones represented by a lumped thermal capacitance, which particularly helps minimize overheating in southern rooms during clear days with significant expected solar gains.

In contrast, an automated approach attempts to construct the most definitive structure through an iterative process; Bacher and Madsen (2011) defined the most accurate structure with a forward procedure that iteratively compares more complex structures to a base structure and tested it on an experimental setup; Prívara et al. (2012) proposed and validated (with surrogate data from TRNSYS) a two-stage procedure that first selects the most relevant inputs and then identifies the order which yields desirable accuracy; Wang et al. (2019) proposed and validated (with data from a real low-energy house) a procedure that starts with a very complex structure and iteratively deletes parameters based on the asymptotic confidence intervals of their estimates; Leprince et al. (2022) presented a methodology that automatically selects the best model structure from a number of pre-set structures; and, Vallianos et al. (2022) proposed and validated (with data from an experimental house) a methodology for multi-zone model generation that derives the most accurate structure by iteratively adding or removing model parameters.

Candanedo et al. (2022) have defined a Control-oriented Archetype Model (CAM) as a gray-box model that provides a generic representation of common zone/building types and, thus, enough information to evaluate the effect of various control sequences in short-term, helping to make near-optimal decisions. Vallianos et al. (2023) used the low-order CAMs, with a heuristic approach, from Abtahi et al. (2022a) and the multi-zone CAM, with an automated approach, from Vallianos et al. (2022) in an online Economic MPC (EMPC) framework and concluded that although low-order CAMs are faster to create and calibrate, achieving satisfactory accuracy requires more information for data processing and aggregation. On the other hand, the multi-zone CAM is fully automatic and is calibrated directly from data from smart thermostats, but it requires significantly more computational resources, which increase exponentially as the timestep of the model decreases.

Grid operators and control firms are increasingly promoting near-optimal practices to foster effective participation in DR programs, creating a “win-win” scenario for the grid and the customer. A well-designed DR program encourages grid-friendly behaviors without jeopardizing occupants’ comfort and causing technical or security issues; conversely, a poorly-designed program credits customers only at the expense of their comfort, leading to reduced participation. To ensure energy equity and encourage participation, it is essential to weigh the magnitude of potential response against the customers’ marginal benefits in a DR program. Therefore, this paper presents a methodology to develop CAMs and use them in an EMPC algorithm to simulate optimal heating load management in response to a recently introduced DR program for Québec’s residential sector; the methodology is then evaluated through a case study wherein the sensitivity of the response to weather forecast uncertainties is analyzed.

## Methodology

Equation (1) is the differential equation governing the heat balance at any node *n* in a thermal RC network, where *C*_*n*_ is the thermal capacitance (J/°C), *R_n,m_* is the thermal resistances (°C/W) in heat exchange with any adjacent node *m*, *T*_*n*_ is the node’s temperature (°C), and *Q*_*n*_ is the heat flow (W) to/from the node. Equation (2) represents an explicit finite difference temporal discretization of equation (1), where *k* and *k* + 1 denote the current and next time steps, respectively, and *δt* is the interval between them (s). The heat flow is disaggregated into controlled electric heat input (*Q_e,n_*), uncontrolled solar heat gains (*Q_s,n_* *=* *α_n_G*), where *G* is the node’s effective irradiance (W/m^2^) and *α*_*n*_ is the equivalent solar aperture (m^2^), and other uncontrolled internal gains (*Q_o,n_*); this equation serves as an explicit model to predict the node’s temperature at the next time-step, given its current temperature and valid parameter values (θ*). Algorithm 1 explains the procedure for CAM parametrization at every prediction horizon (*N*_pre_).

(1) $$C_{n}\frac{dT_{n}}{\mathrm{dt}}=\sum_{m}\left(T_{m}-T_{n}\right)/R_{n,m}+Q_{n}$$

(2) $$T_{n}^{k+1}=\frac{\delta t}{{C_{n}}^{k}}\left(\frac{\sum_{m}\left(T_{m}^{k}-T_{n}^{k}\right)}{{R_{n,m}}^{k}}+Q_{e,n}^{k}+\alpha_{n}G^{k}+Q_{o,n}^{k}\right)+T_{n}^{k};\delta t\leq \min\left(\frac{C_{n}}{\sum_{m}\frac{1}{R_{n,m}}}\right)$$

(3) $$J_{\mathrm{Param}.}=\sum_{n}\omega_{n}\sqrt{\sum_{k=0}^{{N_{\mathrm{train}}}^{*}-1}\sum_{j=0}^{N_{\mathrm{pre}}}\left(\phi_{k,j}{\left({T_{n}}^{k+j}-{{\hat{T}}_{n}}^{k+j|k}\right)}^{2}\right)}$$

Next, the parametrized (recalibrated) CAM is used in a robust EMPC algorithm to minimize space-heating costs and deviation of indoor air temperature from the reference setpoint (*D*). The latter is incorporated into the objective function as a soft constraint with a weighting factor (*P*_*t*_) that balances the cost of electricity against its *equivalent cost*. Algorithm 2 explains the procedure for EMPC at every control horizon (*N*_con_).

(4) $$J_{EMPC}=\sum_{n}\mu_{n}(\sum_{k=0}^{N_{pred}-N_{con-1}}\sum_{j=0}^{N_{con}}\left(\xi_{k,j}\delta t\left(P_{e}^{k*j}({\bar{\bar{Q}}}_{e,n}^{k*j}/\eta_{e})*P_{t}^{k*j}D_{n}^{k*j}\right))\right)$$

CAMs may incorporate nodes for capturing the long-term inertia associated with the *effective-envelop mass* in indoor thermal dynamics, the temperature at which is a *hidden state* as it is not directly measurable but influences the *observed states*, that is, the temperature at nodes designed to capture the short-term inertia associated with the *effective-air mass* in indoor thermal dynamics; a Moving Horizon Estimator (MHE) (Alessandri et al., 2008) leverages the calibrated model, electric heating input records, and weather data from the last *κN*_pre_ steps to initialize the hidden states within the algorithm.

## Case study

### Québec’s context

Québec features three distinct climate regions according to the updated Köppen-Geiger climate classification world map (Peel et al., 2007), which are listed in Table 1. Projected to surpass 8.8 million by the end of 2023 (Statistique Québec, 2022), more than 80% of the province’s population reside in an approximate corridor of 300 km long and 100 km wide extending from Greater Montréal (the province’s most populous metropolitan area) to Québec City (the provincial capital) with a humid continental climate (Statistics Canada, 2021). The remaining population lives in scattered communities under subarctic and arctic climates, engaging in specific activities such as forestry, fishing, and mining (Britannica, n.d.).

**Table 1.** Québec’s climate regions and characteristics based on the updated Köppen-Geiger climate classification (Peel et al., 2007).

Regions	Latitude range	Characteristics	Summers	Winters
Southern and south-western Québec	45–51 °N	Humid continental	Warm to occasionally hot and humid	Cold and snowy
Central Québec	51–58 °N	Subarctic	Short and warm	Long, very cold, and snowy
Northern Québec	≤58 °N	Arctic	Very short and cool	Very long, extremely cold and covered with snow

Unsupervised classification of historical weather data yields typical daily patterns across various seasons. *K*-means clustering algorithms are widely used to partition one dataset into *K* (a pre-defined number) unlabeled datasets, aiming to maximize intra-similarity within each dataset while minimizing inter-similarity across different datasets, with each dataset represented by its centroid (mean of all the assigned data points).

The Standard Hartigan-Wong *K*-means clustering algorithm (Hartigan and Wong, 1979) is applied to classify historical weather data of Trois-Rivières from November 2015 to November 2023. Located roughly at the center of Québec’s populous corridor, it serves as an ideal location for identifying daily profiles of ambient air temperature and solar radiation during typical cold and very cold days in southern and south-western Québec with a humid continental climate. Historical weather is retrieved from the SIMEB (in French, SIMulation Energétique des Bâtiments) website (SIMEB, 2023), which provides (among others) ambient air temperature and Global Horizontal Irradiance (GHI) in hourly intervals from several weather stations in Québec; hourly data is interpolated to 15-min intervals using linear interpolation for temperature and backfilling for irradiance.

**Table:** 

	**Algorithm 1.** CAM parametrization		**Algorithm 2.** EMPC.
	**Inputs:** • CAM ▪ heuristically selected structure (*θ* *=* *[R_n,m_, C_n_, α_n_]*) ▪ initial parameter values (*θ*_*0*_) ▪ physical boundaries for parameter values ( $\bar{\theta }$ , * $\underline{\omega }$ *) • Historical records of ▪ indoor air temperature (*T*_*i*_) ▪ electric heating input (*Q_e,i_*) ▪ ambient air temperature (*T*_*out*_) ▪ effective irradiance (*G*)		**Inputs:** • CAM ▪ heuristically selected structure (*θ* *=* *[R_n,m_, C_n_, α_n_]*) ▪ valid parameter values (θ*) • Uncontrolled inputs for the next prediction horizon ▪ forecast of ambient air temperature ( $\tilde{T}$ _*out*_) ▪ forecast of effective irradiance ( $\tilde{G}$ ) ▪ reference setpoint (*T*_*ref*_) ▪ price of electricity (*P*_*e*_) ▪ equivalent price of deviation from the reference setpoint (*P*_*t*_)
	**Output:** • CAM valid parameter values (θ*)		**Output:** • Optimal electric heating input for the next control horizon ( ${\bar{\bar{Q}}}_{e}$ )
	**Steps:**		**Steps:**
1	Aggregate historical records of indoor air temperature and electric heating input (*T*_*i*_ is the indoor air temperature recorded by the smart thermostat and *C_e,i_* is the electric heating capacity it controls; also, *Q_e,i_* is the recorded electric heating input). $T_{n}=\sum_{i}C_{e,i}T_{i}/\sum_{i}C_{e,i};Q_{e,n}=\sum_{i}Q_{e,i}$	1	Create the indoor temperature and the optimal electric heating input matrices for the next prediction horizon. $\hat{T}={[]}_{N_{\mathrm{pre}}N_{\mathrm{pre}}};{\bar{\bar{Q}}}_{e}={[]}_{N_{\mathrm{pre}}N_{\mathrm{pre}}}$
2	Create and initialize the indoor temperature matrix for multi-step ahead prediction ( κ is the near-optimal ratio of training data length to prediction horizon, and $\hat{T}$ ^u,v^ is the model’s predictions of *v* step ahead at *u*^th^ time-step). $N_{\mathrm{train}}=\kappa .N_{\mathrm{pre}};{N_{\mathrm{train}}}^{*}=(\kappa -1)N_{\mathrm{pre}}$ $\hat{T}={[]}_{{N_{\mathrm{train}}}^{*}N_{\mathrm{pre}}};{\hat{T}}^{0,0}=T^{0}$	2	Minimize the model’s multi-step ahead prediction of space-heating costs and the equivalent cost of deviation from the reference setpoint; compute the sequence of optimal electric heating input { ${\bar{\bar{Q}}}_{e,n}$ ^0^, ${\bar{\bar{Q}}}_{e,n}$ ^1^, …, * ${\bar{\bar{Q}}}_{e,n}$ *^Ncon^} (μ_*n*_ is the spatial weight for each node and *ξ*_*k,j*_ is the temporal weight for each time-step: a decay function to emphasize the importance of nearer term predictions over predictions towards the end of the prediction horizon; *η*_*e*_ is the efficiency of converting electricity to heat and υ_k,j_ is the synthetic noise to account for internal gains; and Q_e,n,max_ is the nodes’s electric heating capacity). $\underset{\left\{{{\bar{\bar{Q}}}_{e,n}}^{:N_{\mathrm{con}}}\right\}}{\min}J_{\mathrm{EMPC}}$ *s.t*: ${\hat{T}}_{n}^{k*j|k}\;=\frac{\delta t}{C_{n}}(\sum_{m\neq {\tilde{T}}_{out}}\left(\frac{{\hat{T}}_{m}^{k*j-1|k}-{\hat{T}}_{n}^{k*j-1|k}}{R_{n,m}}\right)\;*\frac{{\tilde{T}}_{out}^{k*j-1}-{\hat{T}}_{n}^{k*j-1|k}}{R_{n,out}}*Q_{e,n}^{k*j-1}*\alpha_{n}{\tilde{G}}^{k*j-1}*{\bar{\bar{Q}}}_{k,j})*{\hat{T}}_{n}^{k*j-1|k}$ $\theta =\theta^{*}$ $0<{\bar{\bar{Q}}}_{e,n}<Q_{e,n,\max}$ $\xi_{k,j}=e^{-{ϒ}_{2}(k+j)};{ϒ}_{2}>0$ $D_{n}=\left\{ \begin{array}{c} T_{\mathrm{ref}}-\hat{T}\;;\hat{T}<T_{\mathrm{ref}}\\ 0\;;T_{\mathrm{ref}}\leq \hat{T}\leq T_{\mathrm{ref}}+\bar{D}\\ \hat{T}-T_{\mathrm{ref}}\;;T_{\mathrm{ref}}+\bar{D}<\hat{T} \end{array}\right.$
3	Minimize the model’s multi-step ahead prediction error (*ω*_*n*_ is the spatial weight for each node and *ϕ*_*k,j*_ is the temporal weight for each time-step: a decay function to emphasize the importance of nearer term predictions over predictions towards the end of the prediction horizon; υ_k,j_ is the synthetic noise to account for internal gains). $\underset{\theta }{\min}J_{\mathrm{Param}.}$ s.t: ${\hat{T}}_{n}^{k*j|k}\;=\frac{\delta t}{C_{n}}(\sum_{m\neq T_{out}}\left(\frac{{\hat{T}}_{m}^{k*j-1|k}-{\hat{T}}_{n}^{k*j-1|k}}{R_{n,m}}\right)*\frac{T_{out}^{k*j-1}-{\hat{T}}_{n}^{k*j-1|k}}{R_{n,out}}\;*\;Q_{e,n}^{k*j-1}*\alpha_{n}G^{k*j-1}*\upsilon_{k,j})*{\hat{T}}_{n}^{k*j-1|k}$ $\underline{\theta_{n}}<\theta_{n}<\bar{\theta_{n}}$ $\phi_{k,j}=e^{-{ϒ}_{1}\left(k+j\right)};{ϒ}_{1}>0$ $\delta t\leq \min\left(\frac{C_{n}}{\sum_{m}\frac{1}{R_{n,m}}}\right)$		

**Figure 1.**
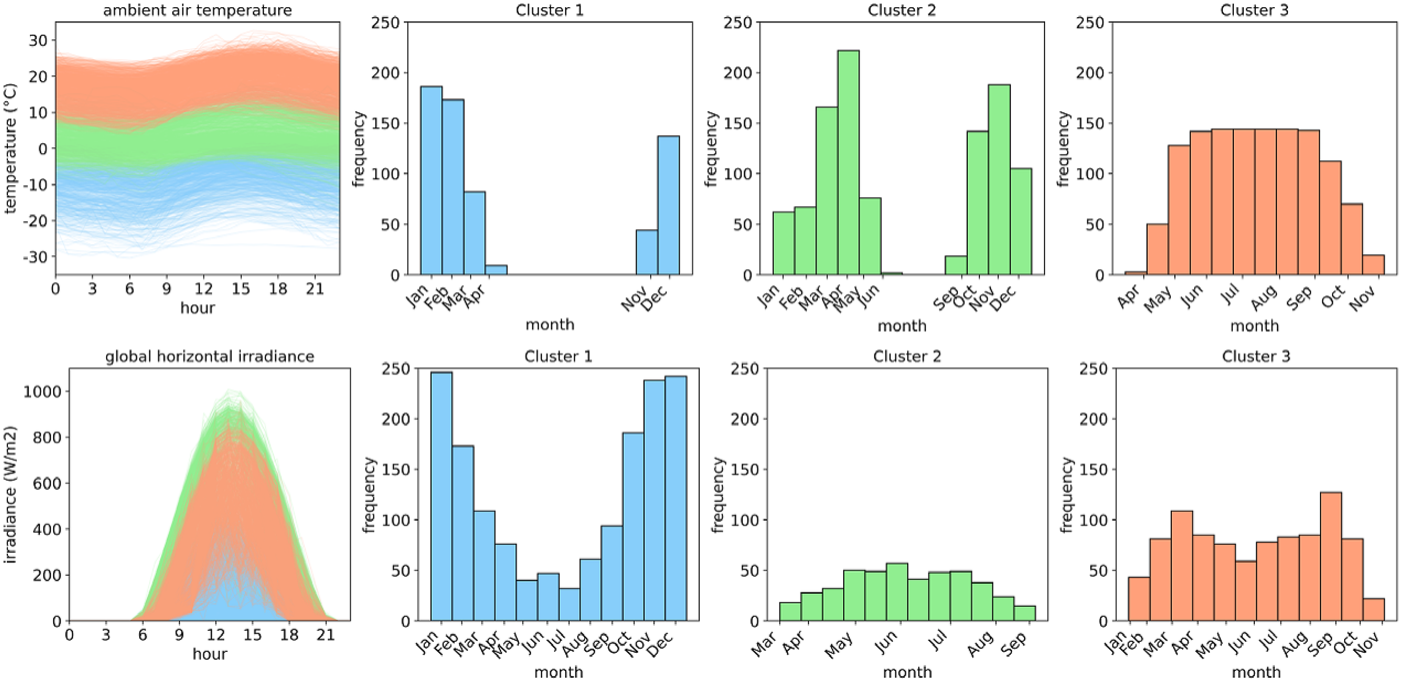
Classification of daily profiles of ambient air temperature and global horizontal irradiance using *K*-means clustering.

Figure 1 illustrates the results of historical weather data clustering. The top left plot classifies daily ambient air temperature profiles into three clusters, while the subsequent plots display the annual distribution for each cluster, and the bottom row provides corresponding information for daily GHI profiles. This clustering offers insights into typical daily profiles throughout the year, particularly focusing on the cold period (blue plot). Ambient air temperature and GHI have naturally different annual distributions due to their distinct seasonal and diurnal patterns.

The Québec residential sector contributes 18% of the province’s total energy consumption, with electric space heating expectedly accounting for approximately two-thirds of this share (Whitmore and Pineau, 2023), designating it as the primary end-use to shape the province’s demand profile and put the grid under stress on very cold weekday mornings and evenings (before and after a typical work schedule). In such a context, the high penetration of decentralized electric baseboard heaters in the province’s residential sector can be leveraged to activate a remarkable energy flexibility potential due to their capacity for individual control according to purpose and occupants’ preferences.

To incentivize off-peak consumption, Hydro-Québec, the public utility managing the generation, transmission, and distribution of electricity in Québec, has recently launched a DR program consisting of a static ToU tariff (rate Flex-D) (Hydro-Québec, 2022) and a smart home service with tailored instructions for reducing consumption through their subsidiary, Hilo. This program allows homeowners to actively reduce their cost of electricity consumption by shifting their load to off-peak periods and, as a result, relieve the pressure on the grid. Table 2 explains, in detail, the structure of rate Flex-D.

**Table 2.** Structure of rate Flex-D: peak demand events may transpire on weekdays from December 1 to March 31, 6:00–9:00 and/or 16:00–20:00, with a projected range of 25–35 events per year (Hydro-Québec, 2022).

Period	April 1 to November 30	December 1 to March 31
System access charge for each day in the consumption period	43.505 ȼ	43.505 ȼ
Price applicable to energy consumed up to 40 kWh times the number of days in the consumption period (first tier)	6.509 ȼ/kWh	4.582 ȼ/kWh
Price applicable to remaining energy consumed (second tier)	10.041 ȼ/kWh	7.880 ȼ/kWh
Price applicable to energy consumed during peak demand events	N/A	53.526 ȼ/kWh

### Simulation inputs

**Figure 2.**
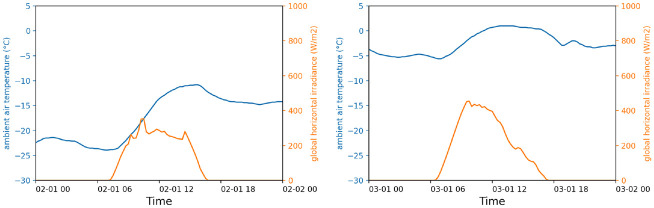
Perfect historical weather of two weekdays as uncontrolled input for EMPC simulation: February 1, 2023 (left) and March 1, 2023 (right).

Perfect (no uncertainty) historical weather of two weekdays in winter 2023 is used as uncontrolled input to simulate optimal heating load management in response to this DR program. The first day, Wednesday, February 1, was among the 5% coldest days observed between 2015 and 2023 and featured both morning and evening peak demand events, while the second day, Wednesday, March 1, was a typical cold day with only a morning peak demand event. Figure 2 shows the uncontrolled weather input for both days.

In situ measurements and metadata from an unoccupied research house 35 km north-west of Trois-Rivières are used to develop a CAM for this case study. It is a detached two-storey house with an excavated, uninsulated basement and an attached garage, measuring 7.6 m × 7.9 m (60 m^2^ footprint) with a floor height of 3 m. The first floor consists of the living room, dining room, kitchen, and a small powder room, while the second floor has three bedrooms and a full bathroom. The house is a representative Québec residence, with R-20 insulation for the walls, R-30 insulation for the roof,^1^ and a total of 19 m^2^ of fenestration consisting of vinyl-framed double-glazing windows with an air gap. It is oriented 35° west of south and is heated with an electric baseboard (*η*_*e*_ = 1) in each room controlled by an individual smart thermostat.

**Figure 3.**
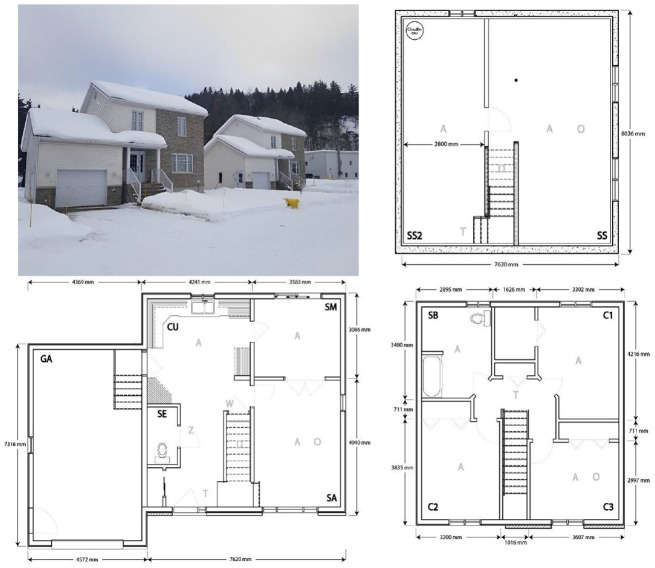
Hydro-Québec’s Experimental House for Building Energetics (EHBE) and its floor plans: the basement (top right), the first floor (bottom left), and the second floor (bottom right).

Measurements consist of perfect (no uncertainty) smart thermostat records of indoor air temperature, heating setpoint, and electric heating input, with 15-min temporal resolution. A Raspberry Pi 4 serves as an access point (node) within the house Internet-of-Things (IoT) network and directly communicates with the smart thermostats, collecting and storing their data in a local database before transferring to an offline server. The layout of the house is shown in Figure 3, and Table 3 shows the location and installed capacity for each baseboard heater.

**Table 3.** Location and installed capacity of each electric baseboard heater.

Abbreviation	Room	Location	Installed heating capacity (kW)
SS	Basement 1	Basement	2
SS2	Basement 2	Basement	2
CU	Kitchen	First floor	1.5
SM	Dining room	First floor	1.25
SA	Living room	First floor	1.5
SE	Powder room	First floor	–
C1	Bedroom 1	Second floor	1.25
C2	Bedroom 2	Second floor	1.25
C3	Bedroom 3	Second floor	1.25
SB	Bathroom	Second floor	1
Total	–	–	13

The authors have acquired insights from a preceding study (Vallianos et al., 2023), suggesting that the zoning-by-floor approach in Abtahi et al. (2022a) (6R3C network), wherein a single lumped capacitance represents an entire floor in a house, may not accurately capture the building’s thermal dynamics, particularly in free-floating conditions, during which the indoor air discharges considerably faster than the building’s effective thermal mass, depicting it unrealistic to combine the two into one lumped capacitance; therefore, for this case study, an 11R6C network is developed with a heuristic zoning-by-floor approach, where each floor is a separate thermal zone represented by two lumped thermal capacitances: one for capturing the short-term inertia associated with the effective-air mass and another for capturing the long-term inertia associated with the effective-envelop mass. Since the building is unoccupied, the only heat sources considered are the controlled electric heating input and the uncontrolled solar heat gains.

**Figure 4.**
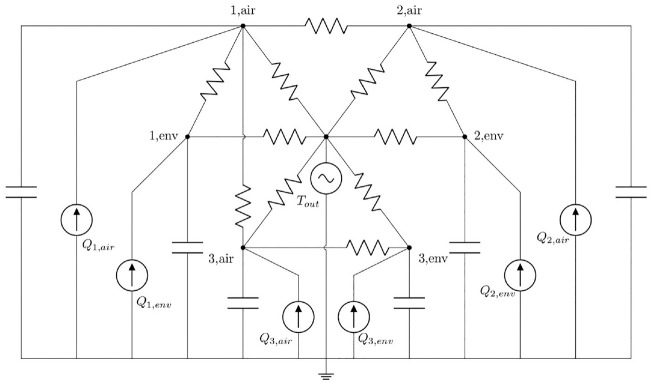
Case study CAM structure: 11R6C network with the heuristic zoning-by-floor approach.

This archetype network is shown in Figure 4, and its information is listed in Table 4.

**Table 4.** Case study CAM information: *Q*_*e*_ is the electric heat input, and *Q*_s_ is the solar heat gain.

Node (*n*)	Description	Adjacent nodes (*m*)	Heat source (*Q*_*n*_)
1,air	First-floor indoor air	1,env and 2,air.	*Q*_e1,air_ + *Q*_s1,air_
2,air	Second-floor indoor air	2,env and 1,air	*Q*_e2,air_ + *Q*_s2,air_
3,air	Basement indoor air	3,env and 1,air	*Q*_e3,air_ + *Q*_s3,air_
1,env	First-floor effective thermal mass	1,air	*Q*_s1,env._
2,env	Second-floor effective thermal mass	2,air	*Q*_s2,env._
3,env	Basement effective thermal mass	3,air	*Q*_s3,env._
*T*_out_	Ambient air temperature	All the other nodes	N/A

Algorithm 1 recalibrates the CAM at the beginning of each day (*N*_pre_ = 24 h), using the records of the past 3 days for training (*N*_train_* = 72 h), and initializes with the calibrated values from the previous execution (θ^0^ = θ^*,−1^). The training data for each day includes both upward and downward temperature variations within the comfort range of 18–24°C, ensuring a robust representation of indoor thermal dynamics under varying conditions. Calibration results for both days are summarized in Tables 5 and 6.

**Table 5.** Case study CAM parameters: valid parameter values for simulation on February 1; *R* (°C/W), *C* (MJ/°C), and α (m^2^).

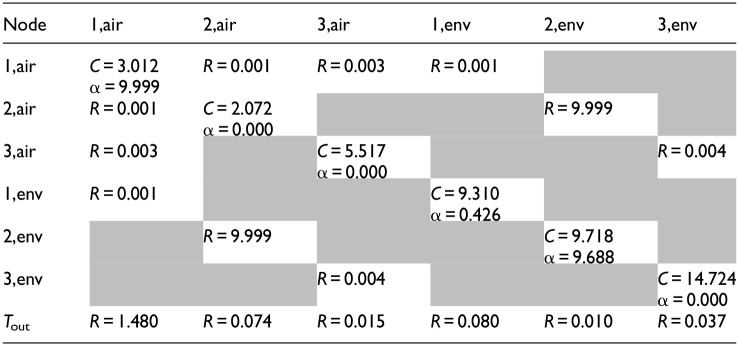

**Table 6.** Case study CAM parameters: valid parameter values for simulation on March 1; *R* (°C/W), *C* (MJ/°C), and α (m^2^).

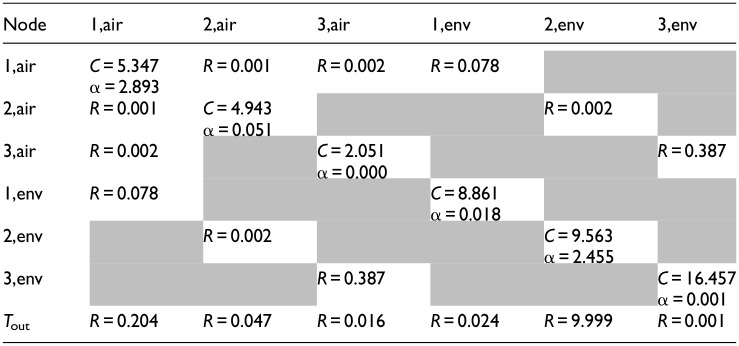

The capacitance values (MJ/°C) reflect the building’s overall ability to store thermal energy. The values obtained from calibration are consistent with the expected physical properties of building materials and the volume of indoor air, with indoor air nodes corresponding to the air volume in each zone and effective thermal mass nodes representing the heat storage capacity of building materials, showing similar values for the first and second floors and higher values for the basement, consistent with its heavier construction materials. The variations in capacitance values between different days indicate changes in environmental conditions and operational factors, providing an accurate and adaptable model.

The resistance values (°C/W), represent the thermal resistance between different nodes, characterizing the heat transfer within the building. The variability in resistance values between February 1 and March 1 reflects distinct responses to various weather conditions; notably, the resistances toward the ambient air temperature show significant variations between the two cases, with lower values on the colder day (February 1) and higher values on the milder day (March 1).

The solar aperture values (m^2^), represent the effective area through which solar radiation contributes to the building’s heat gain. These values are distributed between the air and envelope nodes to reflect the building’s design and material properties. Higher solar aperture values for air nodes indicate significant direct solar gains affecting indoor air temperature, while envelope nodes capture the thermal inertia associated with solar heat absorbed by the building materials. The presence of α values on both air and envelope nodes is justified by the need to account for both immediate and delayed effects of solar radiation. The differences in solar aperture values between the 2 days underscore the model’s sensitivity to varying solar conditions, ensuring accurate predictions of the building’s thermal response to solar gains.

**Figure 5.**
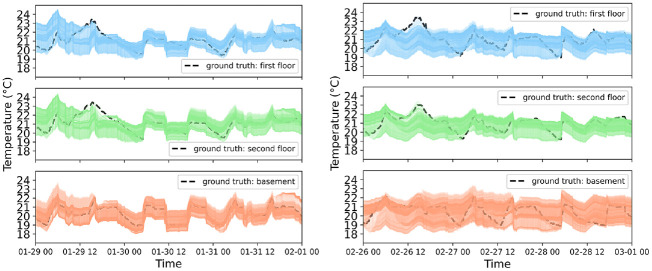
Case study CAM performance: indoor air temperature records (ground truth) versus the CAM’s 24 h—ahead predictions—February 1, 2023 (left) and March 1, 2023 (right).

Figure 5 compares the actual indoor air temperature (ground truth) in a thick dashed black line against the CAM’s 24 h-ahead predictions in thin colored lines; at each timestep, a thin colored line represents the prediction of indoor air temperature starting from that moment and projecting forward for 24 h based on previous predictions. Nearer-term predictions tend to be more accurate and closely align with the ground truth compared to predictions further into the future, reflecting the accumulation of prediction errors and uncertainties over time, which leads to more significant divergence from the actual observed behavior as the prediction horizon extends. To address this, a decay function is introduced to underscore the significance of nearer-term predictions over those toward the end of the prediction horizon. Therefore, the discrepancy pertains to the variance between further-term predictions and the ground truth. However, it’s worth noting that the effect of this discrepancy is mitigated in the MPC.

Subsequently, Algorithm 2 uses the recalibrated CAM every 6 h to compute the sequence of optimal electric heating input for the next 6 h (*N*_con_ = 6 h). The daily reference setpoint profile is derived from a recent study (Henao et al., 2018), wherein the analysis of data from 300 smart thermostats situated in 30 houses in Trois-Rivières indicated that the most probable times for heating setpoint adjustments are between 5:00 and 6:00 for morning set-ups, at 8:00 for morning set-backs, between 16:00 and 17:00 for evening set-ups, and at 22:00 for evening set-backs; this study also reported that the most likely heating setpoint is 21°C. Therefore, the daily reference setpoint profile in the first and second floors is established with a high of 21°C, a low of 19°C, a morning set-up at 5:00, a set-back at 8:00, an evening set-up at 17:00, and a set-back at 22:00; in the basement, the setpoint profile is a constant 17°C.

Indoor air temperatures deviating below the reference setpoint or exceeding it by more than 3° (*D* = 3°C) are penalized with a default equivalent cost of *P*_*t*_ = 275 ȼ/°Ch, which implies that 1 °Ch of deviation from the reference setpoint *costs* approximately as much as 5 kWh of electricity consumption during the peak-demand events and 55 kWh during off-peak periods. All the zones are equally weighted (ω_n_ = μ_n_ = 1), while nearer-term predictions are emphasized over predictions toward the end of the prediction horizon with a gentle decay factor (ϒ_1_ = ϒ_2_ = 0.01). The reference BaU operation is assumed to reactively maintain the indoor air temperature at the reference setpoint without considering the price of electricity; therefore, it is highly expected that the latter closely tracks the former.

**Figure 6.**
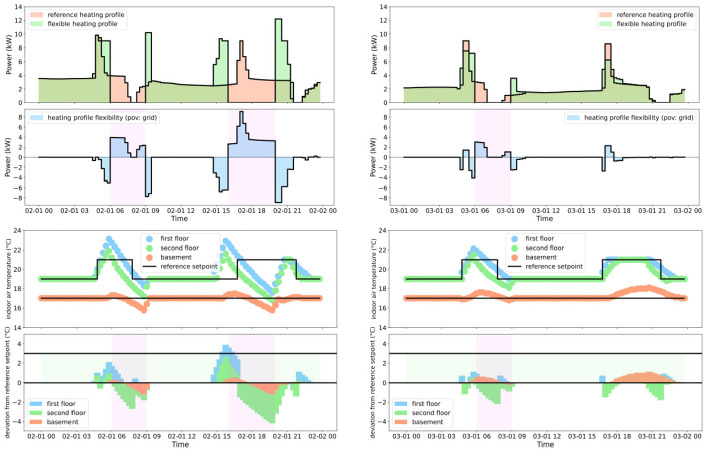
Case study EMPC performance: reference heating demand profile versus flexible heating demand profile (top); daily reference setpoint profile versus the predicted indoor air temperature (bottom)—February 1, 2023 (left) and March 1, 2023 (right).

Figure 6 (top plot) depicts the flexible cost-optimal heating demand profile in comparison to the reference heating demand profile, along with their difference (latter−former); this figure (bottom plot) also presents the daily reference setpoint profile versus the predicted indoor air temperature, along with their difference (latter−former). The horizontal green band indicates the range in which deviation from the reference setpoint incurs no penalties, while deviations outside this range are linearly penalized; also, the vertical violet bands highlight peak demand events, where electricity is more expensive than in the white areas. EMPC leverages the lower electricity price to charge the building’s thermal mass during off-peak hours and later relaxes the heating system unless the indoor air temperature approaches the lower limit of the non-penalized range; this significantly reduces heating demand during peak demand events, leading to substantial cost savings.

Finally, the average demand deviation from *t* to *t* + Δ*t* is calculated using equation (5) (Athienitis et al., 2020), where *P*_e,ref_ and *P*_e,flex_ are the reference and the flexible profiles, respectively; equation (6) calculates the average deviation of indoor air temperature from the reference setpoint for the same period.

(5) $$\Delta P_{\mathrm{avg}}(t,\Delta t)=\left(\int_{t}^{t+\Delta t}P_{e,\mathrm{ref}}(t)\mathrm{dt}-\int_{t}^{t+\Delta t}P_{e,\mathrm{flex}}\left(t\right)\mathrm{dt}\right)/\Delta t$$

(6) $$D_{\mathrm{avg}}(t,\Delta t)=\left(\sum_{n=1}^{N}\left(\int_{t}^{t+\Delta t}D_{n}(t)\mathrm{dt}\right)/\Delta t\right)/N$$

The simulation is repeated with two different values for the equivalent price of deviation from the reference setpoint, 1375 and 55 ȼ/°Ch, and the results are summarized in Tables 7 and 8.

**Table 7.** Case study EMPC performance: average deviation from the reference BaU operation; Δ*P*_avg_ (kW) and *D*_avg_ (°C).

Day	February 1			March 1		
*P*_*t*_	1375 ȼ/°Ch	275 ȼ/°Ch	55 ȼ/°Ch	1375 ȼ/°Ch	275 ȼ/°Ch	55 ȼ/°Ch
00:00–06:00	Δ*P*_avg_ = −0.3 *D*_avg_ = 0.0	Δ*P*_avg_ = −0.5 *D*_avg_ = 0.1	Δ*P*_avg_ = −0.5 *D*_avg_ = 0.1	Δ*P*_avg_ = −0.3 *D*_avg_ = 0.0	Δ*P*_avg_ = −0.3 *D*_avg_ = 0.0	Δ*P*_avg_ = −0.2 *D*_avg_ = −0.0
06:00–09:00	Δ*P*_avg_ = 1.2 *D*_avg_ = −0.1	Δ*P*_avg_ = 2.5 *D*_avg_ = −0.5	Δ*P*_avg_ = 2.5 *D*_avg_ = −0.6	Δ*P*_avg_ = 1.1 *D*_avg_ = −0.2	Δ*P*_avg_ = 1.1 *D*_avg_ = −0.3	Δ*P*_avg_ = 1.1 *D*_avg_ = −0.3
09:00–16:00	Δ*P*_avg_ = −0.4 *D*_avg_ = 0.0	Δ*P*_avg_ = −1.5 *D*_avg_ = 0.2	Δ*P*_avg_ = −1.3 *D*_avg_ = 0.1	Δ*P*_avg_ = −0.2 *D*_avg_ = −0.0	Δ*P*_avg_ = −0.2 *D*_avg_ = −0.0	Δ*P*_avg_ = −0.2 *D*_avg_ = −0.0
16:00–20:00	Δ*P*_avg_ = 1.1 *D*_avg_ = −0.2	Δ*P*_avg_ = 4.1 *D*_avg_ = −0.9	Δ*P*_avg_ = 4.1 *D*_avg_ = −1.2	Δ*P*_avg_ = 0.0 *D*_avg_ = 0.1	Δ*P*_avg_ = 0.0 *D*_avg_ = 0.0	Δ*P*_avg_ = 0.8 *D*_avg_ = -0.5
20:00–24:00	Δ*P*_avg_ = −0.8 *D*_avg_ = 0.0	Δ*P*_avg_ = −2.2 *D*_avg_ = −0.3	Δ*P*_avg_ = −2.3 *D*_avg_ = −0.4	Δ*P*_avg_ = −0.0 *D*_avg_ = 0.1	Δ*P*_avg_ = −0.0 *D*_avg_ = 0.1	Δ*P*_avg_ = −0.5 *D*_avg_ = −0.3

**Table 8.** Case study EMPC performance: daily average deviation of indoor air temperature from the reference setpoint versus percentage of daily heating cost reduction.

Day	February 1			March 1		
*P*_*t*_	1375 ȼ/°Ch	275 ȼ/°Ch	55 ȼ/°Ch	1375 ȼ/°Ch	275 ȼ/°Ch	55 ȼ/°Ch
Davg (°C)	0.0	−0.2	−0.3	0.0	−0.0	−0.2
Cost reduction (%)	−124.2^a^	30.9	31.8	29.9	30.1	32.2

a A negative cost reduction indicates an increase in heating costs compared to the reference scenario.

Table 7 shows a trade-off between *P*_*t*_ and Δ*P*_avg;_ the more deviation of indoor air temperature from the reference setpoint is penalized (higher *P*_*t*_), the less EMPC uses the building’s thermal mass for pre-heating during off-peak hours and free-floating during peak demand events (lower Δ*P*_avg_). As per Table 8, EMPC achieves an approximately 30% reduction in daily heating cost with a minimal average deviation of indoor air temperature from the reference setpoint.

However, introducing a large value for *P*_*t*_ may result in an increase in heating cost; using *P*_*t*_ = 1375 ȼ/°Ch for the simulation on February 1, the daily heating cost reaches 11.0 $, representing 124% increase compared to the reference daily heating cost of 4.9 $, due to heating during peak demand events, where electricity is 12 times more expensive.

The simulation is repeated, incorporating weather forecast uncertainty for the next control horizon as a noise added to perfect historical weather, with a maximum of 1.5% for the ambient air temperature and a maximum of 15% for the global horizontal irradiance (Voyant et al., 2017); the results indicate that the most influential uncontrolled input directing the performance of EMPC is the structure of rate Flex-D, featuring substantial step changes and rendering the impact of uncertainty in the weather forecast negligible, with less than a 1% difference in predicted cost reduction.

## Conclusion

Grid operators and control firms are increasingly promoting near-optimal practices to foster effective participation in DR programs, creating a “win-win” scenario for both the grid and the customer. To ensure energy equity and encourage participation, it is essential to weigh the magnitude of potential response against the customers’ marginal benefits in a DR program. To address this requirement, this paper presented a methodology to develop CAMs and use them in an EMPC algorithm to simulate optimal heating load management in response to a recently-introduced static ToU tariff for Québec’s residential sector, rate Flex-D; the methodology was then evaluated through a case study.

For the case study, perfect in situ measurements from a two-storey unoccupied research house of Hydro-Québec were used to develop an 11R6C network with a heuristic zoning-by-floor approach, where each floor is a separate thermal zone represented by two lumped thermal capacitances: one for capturing the short-term inertia associated with the effective-air mass and another for capturing the long-term inertia associated with the effective-envelop mass.

Given the building’s unoccupancy, heat sources were assumed to be exclusively the controlled electric heating input and the uncontrolled solar heat gains. Perfect historical weather of two weekdays in winter 2023 was used as uncontrolled input for the simulation; the first day, Wednesday, February 1, was among the 5% coldest days observed between 2015 and 2023 and featured both morning and evening peak demand events; and the second day, Wednesday, March 1, was a typical cold day with only a morning peak demand event only. The model was recalibrated at the beginning of each day, using the records of the past 3 days for training and was initialized with the calibrated values from the previous execution.

Subsequently, it was used in EMPC under rate Flex-D every 6 h to compute the sequence of optimal electric heating input for the next 6 h. The daily reference setpoint profile in the first and second floors was established with a high of 21°C, a low of 19°C, a morning set-up at 5:00, a set-back at 8:00, an evening set-up at 17:00, and a set-back at 22:00; in the basement, the setpoint profile was a constant 17°C. Indoor air temperatures deviating below the reference setpoint or exceeding it by more than 3°C were penalized, and the reference operation was assumed to reactively maintain the indoor air temperature at the reference setpoint, without considering the price of electricity.

The daily average deviation of indoor air temperature from the reference setpoint and the percentage of daily heating cost reduction were compared, given different penalization factors (*P*_*t*_); generally, EMPC achieved an approximately 30% reduction in daily heating cost compared to the reference operation, with a minimal average deviation of indoor air temperature from the reference setpoint. Finally, the analysis of the response’s sensitivity to weather forecast uncertainties indicated that the most influential uncontrolled input directing the performance of EMPC is the structure of rate Flex-D, featuring substantial step changes and rendering the impact of uncertainty in the weather forecast negligible, with less than a 1% difference in predicted cost reduction.

## Limitations and future work

The Case Study section primarily serves as a demonstration of using the proposed methodology for recursive day-ahead optimal space-heating load management in the context of the Québec residential sector; the prediction horizon in the case study is 24 h, and at the beginning of every day, the model parameters change, effectively representing a new model iteration (same structure, new parameter values). Therefore, extending the analysis beyond 1 day would not evaluate a unique model but rather multiple iterations of the same structure with varying parameter values. For example, if the analysis were performed over a consecutive 1-week period, it would essentially entail evaluating seven distinct models (one for each day). While this demonstration is limited to 2 days (February 1 and March 1) for the sake of brevity, the methodology is scalable and adaptable to different scenarios. However, incorporating more days into the future analysis offers a more accurate distribution of potential flexibility metrics.

The present study uses fixed spatial and temporal weights in Algorithms 1 and 2, evenly distributing importance among all zones (ω_n_ = μ_n_ = 1) and emphasizing near-term predictions with gentle decay factors (ϒ_1_ = ϒ_2_ = 0.01). These heuristic values have been effective for this specific case study but may not be optimal for all scenarios, potentially limiting the accuracy and adaptability of the predictive model across different building configurations and seasonal variations. Future work involves refining (tuning) these spatial and temporal weights through sensitivity analysis and optimization techniques to better account for the relative importance of each node within the thermal RC network and to adjust the emphasis on predictions over different horizons. This refinement will consider seasonal variations and differing building configurations to enhance model robustness and performance. Fine-tuning these weights precisely improves the accuracy and applicability of the proposed methodology across a broader range of scenarios.

The current study assumes all measurements to be perfect, without considering any measurement uncertainties. This assumption could affect the robustness and accuracy of the predictive model, as real-world data often contain some degree of error. Future research will address this limitation by incorporating realistic measurement uncertainties, providing a more accurate and resilient model that better reflects practical scenarios.

Another limitation is the lack of comparison between the validation metrics and existing standards. This omission limits the contextual understanding of the model’s performance relative to established benchmarks. To enhance the comprehensiveness of future research, validation metrics will be compared against relevant standards, ensuring a clearer assessment of the model’s accuracy and reliability.

Moreover, the study’s findings are based on simulations, which may not fully represent real-world conditions. As a future step, the methodology presented will be implemented in the research house modeled in this paper. This real-world implementation will validate the simulation results and provide practical insights, ensuring the methodology’s applicability and effectiveness in real-world scenarios.

## Appendix

### Nomenclature

#### Acronyms/abbreviations

CAM control-oriented archetype model

DR demand response

EBC energy in buildings and communities

EHBE experimental house of building energetics

EMPC economic model predictive control

GEB grid-interactive efficient buildings

GHI global horizontal irradiance

IEA international energy agency

IoT internet-of-things

KPI key performance indicator

MHE moving horizon estimator

MPC model predictive control

RC resistance-capacitance

RMSE root mean squared error

SIMEB SIMulation Energétique des Bâtiments

#### Subscripts

*e* electric/electricity

*flex* flexible

*i* indoor

*m* node m (adjacent node)

*max* maximum

*n* node n

*o* other

*pre* prediction

*ref* reference

*s* solar

*set* setpoint

*t* thermal

*test* test

*train* train

#### Superscripts

*** validated

*j* *k*-th timestep

*k* *k*-th timestep

#### Greek letters

υ noise

α solar aperture

η efficiency

θ parameter values

ϒ discount factor

ω spatial weight (Algorithm 1)

κ near-optimal ratio of training data length to prediction horizon

*μ* spatial weight (Algorithm 2)

*ξ* temporal weight (Algorithm 2)

*ϕ* temporal weight (Algorithm 1)

#### Latin letters

*C* thermal capacitance (J/°C)

*G* effective irradiance (W/m^2^)

*N* total number of data points

*P* price (ȼ)

*R* thermal capacitance (°C/W)

*T* temperature (°C)

*Q* heating (W)

## Notes

1. R-20 and R-30 indicate the thermal resistance values of the insulation material. R-20 has a thermal resistance of 20 h·ft^2^·°F/Btu (3.52 m^2^·K/W), and R-30 has a thermal resistance of 30 h·ft^2^·°F/Btu (5.28 m^2^·K/W), which are standard measures of insulation effectiveness.
